# Embryonic-Derived *Myb*^−^ Macrophages Enhance Bacterial Clearance and Improve Survival in Rat Sepsis

**DOI:** 10.3390/ijms22063190

**Published:** 2021-03-20

**Authors:** Mirjana Jerkic, Michael L. Litvack, Stéphane Gagnon, Gail Otulakowski, Haibo Zhang, Ori Rotstein, Brian P. Kavanagh, Martin Post, John G. Laffey

**Affiliations:** 1 Keenan Research Centre for Biomedical Science, Unity Health Toronto St. Michael’s, University of Toronto, Toronto, ON M5B 1T8, Canada; Mirjana.Jerkic@unityhealth.to (M.J.); Stephane.Gagnon@unityhealth.to (S.G.); haibo.zhang@unityhealth.to (H.Z.); ori.rotstein@unityhealth.to (O.R.); 2 Translational Medicine Program, Hospital for Sick Children, University of Toronto, Toronto, ON M5G 0A4, Canada; michael.litvack@sickkids.ca (M.L.L.); gail.otulakowski@gmail.com (G.O.); brian.kavanagh@sickkids.ca (B.P.K.); martin.post@sickkids.ca (M.P.); 3 Department of Critical Care Medicine, Hospital for Sick Children, University of Toronto, Toronto, ON M5G 1X8, Canada; 4 Departments of Anesthesia, Physiology and Interdepartmental Division of Critical Care, University of Toronto, Toronto, ON M5S 1A1, Canada; 5 Department of Anesthesia and Critical Care Medicine, Unity Health Toronto St. Michael’s, Toronto, ON M5B 1W8, Canada; 6 Regenerative Medicine Institute (REMEDI) at CÚRAM Centre for Research in Medical Devices, Biomedical Sciences Building, School of Medicine, National University of Ireland Galway, H91 TK33 Galway, Ireland

**Keywords:** *Myb*^−^ peritoneal macrophages, embryonic, rat experimental model, sepsis, phagocytosis, efferocytosis

## Abstract

Peritoneal resident macrophages play a key role in combating sepsis in the peritoneal cavity. We sought to determine if peritoneal transplantation of embryonic *Myb*^−^ “peritoneal-like” macrophages attenuate abdominal fecal sepsis. Directed differentiation of rodent pluripotent stem cells (PSCs) was used in factor-defined media to produce embryonic-derived large “peritoneal-like” macrophages (Ed-LPM) that expressed peritoneal macrophage markers and demonstrated phagocytic capacity. Preclinical in vivo studies determined Ed-LPM efficacy in rodent abdominal fecal sepsis with or without Meropenem. Ex vivo studies explored the mechanism and effects of Ed-LPM on host immune cell number and function, including phagocytosis, reactive oxygen species (ROS) production, efferocytosis and apoptosis. Ed-LPM reduced sepsis severity by decreasing bacterial load in the liver, spleen and lungs. Ed-LPM therapy significantly improved animal survival by ~30% and reduced systemic bacterial burden to levels comparable to Meropenem therapy. Ed-LPM therapy decreased peritoneal TNFα while increasing IL-10 concentrations. Ed-LPMs enhanced peritoneal macrophage phagocytosis of bacteria, increased macrophage production of ROS and restored homeostasis via apoptosis and efferocytosis-induced clearance of neutrophils. In conclusion, Ed-LPM reduced systemic sepsis severity, improved survival and reduced bacterial load by enhancing peritoneal macrophage bacterial phagocytosis and killing and clearance of intra-peritoneal neutrophils. Macrophage therapy may be a potential strategy to address sepsis.

## 1. Introduction

Sepsis is a syndrome characterized by a dysregulated immune response to microbial invasion, which can progress to life-threatening dysfunction of multiple organs [1]. It is a major public health burden in terms of mortality [2,3], economic cost [4], and—in those who survive—long term psychological, cognitive, and physical impairments [5,6]. Sepsis has a mortality rate of 40%, and is implicated in half of all in-hospital deaths in the US [7]. Sepsis survivors continue to have a higher mortality in the 5 years following sepsis [8]. The host immune response to microbial infection is critical, with early phase sepsis characterized by a “hyper-inflammatory” immune response, whereas the later phase of sepsis is often complicated by suppression. Sepsis has no treatment and management remains supportive. Intra-abdominal polymicrobial bacterial infections are among the most common and most severe causes of sepsis [9].

Macrophages, which are present in almost all tissues, coordinate developmental, metabolic, and immunological functions, and thus contribute to the maintenance of immune homeostasis [10]. Macrophage dysfunction plays a key role in the pathogenesis of multiple diseases [11], and therefore, these cells represent attractive therapeutic targets for sepsis. Peritoneal macrophages (PM) are the most abundant population of innate immune cells in the peritoneal cavity and play a pivotal role in the innate immune response to bacterial infection. Two subsets of PMs co-exist in the peritoneal cavity, which exhibit distinct phenotypes, functions, and origins; namely, large peritoneal macrophages (LPMs) and small peritoneal macrophages (SPMs). LPMs appear to be derived from the fetal yolk sac [12], are “constitutively“ present in the peritoneal cavity, and play a key role in immune surveillance and maintenance of tissue homeostasis. Tissue macrophages like LPMs that arise from yolk sac origins (also named primitive macrophages) arise from primitive hematopoiesis. This occurs prior to the developmental arrival of the hematopoietic stem cell (HSCs)—a cell that can give rise to all blood cell lineages—during embryonic blood development. Currently, of the known myeloid lineages, only primitive macrophages and red blood cells have been shown to develop embryonically and independently of the HSC. A master regulator of HSCs is the transcription factor *Myb*, which is crucial for self-renewal [13]. Since primitive macrophages are independent of HSCs, they are also independent of *Myb*. The yolk sac origin of LPMs is indicative of primitive embryonic hematopoeisis, which occurs independently of *Myb* [14,15,16]. Conversely, SPMs originate from HSC-derived bone-marrow myeloid precursors, such as monocytes, and constitute a minor subset in the healthy peritoneal cavity. Like many recruited monocyte-derived macrophages, SPMs appear in large numbers during inflammation and are the major source of inflammatory mediators in the peritoneal cavity during infection.

Given that peritoneal cavity resident macrophages are predominantly LPMs, and are derived from the yolk sac embryologically, we sought to determine the therapeutic potential of intra-peritoneal transplantation of embryologically-derived “LPM-like“ functional macrophages for peritoneal sepsis. We hypothesized that Ed-LPMs would enhance the initial immune response to bacterial sepsis and attenuate local and systemic injury that is often sustained due to exaggerated and poorly controlled immune regulation during sepsis. We have previously demonstrated that similar *Myb*^−^ “alveolar-like“ macrophages derived from pluripotent stem cells (PSCs) have therapeutic benefit in experimental pulmonary transplantation and animal models of acute and chronic airway diseases [17]. Moreover, pluripotent stem cells are a reliable source to generate *Myb*-independent macrophages [18]. In the present studies, we used directed differentiation of rat embryonic stem cells (ESCs) to produce an expandable embryonic-derived “LPM-like“ macrophage (Ed-LPM) phenotype. These studies tested the efficacy and mechanisms of action of intra-peritoneal Ed-LPM therapy in rat polymicrobial abdominal sepsis. We hypothesized that Ed-LPMs would improve survival and enhance bacterial clearance, and investigated the mechanisms underlying these effects.

## 2. Results

### 2.1. Characterization of Embryonic Derived Macrophages (Ed-LPM)

**Figure 1:**
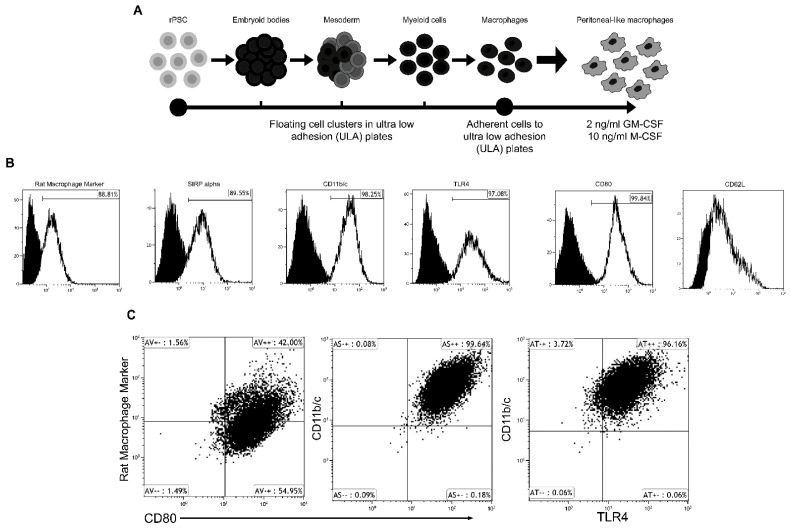
Characterization of embryonic-derived large “peritoneal-like” macrophages (Ed-LPMs). Panel (**A**) represents a schematic of Ed-LPMs derivation from rat embryonic stem cells. ED-LMPs were characterized by flow cytometry for known surface markers (Panel (**B**,**C**)).

Rat Ed-LPMs were derived as previously described [17], whereby pluripotent stem cells were directed through differentiation of primitive mesoderm and hematopoiesis to generate *Myb*^−^ macrophages with the reported efficiency described by Litvack and colleagues [17]. The resultant primitive macrophages were conditioned to peritoneal-like conditions and expanded in 10 ng/mL M-CSF and 2 ng/mL GM-CSF. We performed flow cytometric analysis of the cells for expression of the following LPM surface markers: Rat Macrophage marker; SIRP-alpha; CD11b/c; TLR4; CD80; and the negative marker CD62L. Flow cytometry revealed surface expression of these LPM markers, on the vast majority of cells, including SIRPalpha (88.8%), CD11b/c (98.3%), TLR4 (97.1%), CD80 (99.8%), and a general rat macrophage marker (88.81%) (Figure 1A–C). The Ed-LPMs did not express CD62L. Furthermore, co-staining of Ed-LPMs demonstrated that most cells co-expressed the above indicated markers. This surface expression profile is in agreement with expression arrays from previous publications [15,19].

### 2.2. Efficacy of Ed-LPM in In Vivo Fecal Sepsis

#### 2.2.1. Series 1: Ed-LPM Decreases Severity of Fecal Sepsis

To determine the therapeutic efficacy of Ed-LPMs to reduce the severity of sepsis, we used a model of fecal sepsis to induce injury in rats. Forty one animals were entered into this study, with 6 randomized to Sham + PBS, 6 to Sham + Ed-LPM, 15 to Sepsis + PBS, and 14 to Sepsis + Ed-LPM therapy. Mortality was 0% in the sham animals, 20% in Sepsis + PBS, and 21% in Sepsis + Ed-LPM therapy groups (P = NS). From these animals, we assessed the inflammatory cells, cytokines, and systemic bacterial burden.

##### Inflammatory Cells and Cytokines

**Figure 2:**
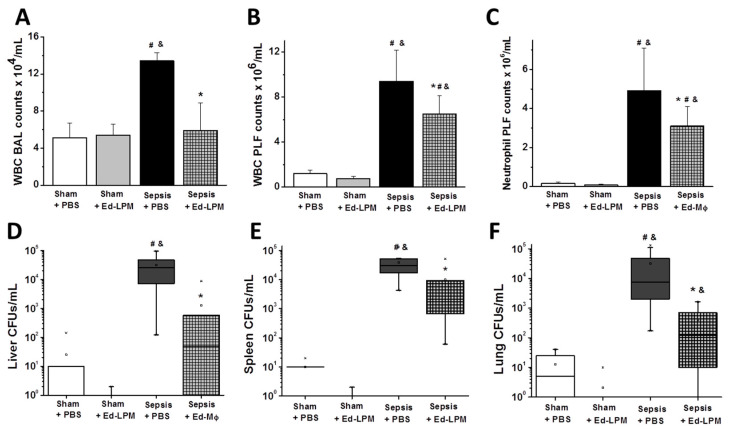
Embryonic derived macrophages (Ed-LPM) decrease severity of fecal sepsis. Ed-LPM therapy attenuated the sepsis induced increase in white blood cell (WBC) infiltration in the broncho-alveolar lavage (BAL) fluid (Panel **A**, *p* = 0.007) and peritoneal lavage (PLF) fluid (Panel **B**, *p* < 0.0001); this effect appeared largely due to reduced neutrophil infiltration (Panel **C**, *p* < 0.0001). Ed-LPM therapy reduced the sepsis induced increase in bacterial load in the liver (Panel **D**, *p* = 0.006), spleen (Panel **E**, *p* = 0.012), and the lung (Panel **F**, *p* = 0.032) *n* = 6 for Sham groups and 10–11 for groups with cecal slurry induced Sepsis; * *p* < 0.05 vs. sepsis + PBS group, ^#^ *p* < 0.05 vs. Sham + PBS group, ^&^ *p* < 0.05 vs. Sham + ED-LPM group.

Ed-LPM therapy attenuated the sepsis induced increase in white blood cell (WBC) infiltration in the BAL fluid (values in 10^4^ CFU/mL in Sepsis + PBS = 13.4 +/− 0.9 vs. Sepsis + Ed-LPM = 5.9 +/− 3, *p* = 0.006) (Figure 2A), and peritoneal lavage (PLF) fluid (values in 10^6^/mL in Sepsis + PBS = 9.4 +/− 2.8 vs. Sepsis + Ed-LPM = 6.5 +/− 1.6, *p* < 0.0001) (Figure 2B). This effect appeared largely due to reduced neutrophil infiltration (values in 10^6^/mL for Sepsis + PBS = 4.9 +/− 2.2 vs. Sepsis + Ed-LPM = 3.1 +/− 1.0, *p* < 0.0001) (Figure 2C). The concentration of the inflammatory cytokine, TNFα, in peritoneal lavage of the Ed-LPM treated group remained similar to that of the sham group, whereas the vehicle treated septic group displayed significantly higher TNFα concentrations (Sham = 34.1 +/− 11.6 pg/mL vs. Sepsis + PBS = 91.2 +/− 55 vs. pg/mL vs. Sepsis + Ed-LPM = 61.3 +/− 18.9, *p* = 0.018) (Figure S1A). Furthermore, Ed-LPM therapy increased IL-10 concentration in PLF in comparison to controls (Figure S1B, *p* < 0.001).

##### Bacterial Burden

Colony forming units (CFUs) of bacteria were assessed in several organs to evaluate systemic bacterial burden. Septic animals treated with Ed-LPM displayed significantly lower bacteria load in comparison to vehicle-treated animals in in the liver (Sepsis + PBS = 3.15 +/− 3.1 × 10^4^ vs. Sepsis + Ed-LPM = 1.3 +/− 2.8 × 10^3^ CFU/mL, *p* = 0.006) (Figure 2D), spleen (values in 10^4^ CFU/mL for Sepsis + PBS =3.9 +/− 3.7 vs. Sepsis + Ed-LPM = 0.98 +/− 1.8, *p* = 0.012) (Figure 2E), and lungs (PFU/mL in Sepsis + PBS = 3.22 +/− 4.9 × 10^4^ vs. Sepsis + Ed-LPM = 4 +/− 5.6 × 10^2^, *p* = 0.032) (Figure 2F).

Taken together, these data suggest that Ed-LPM suppress the inflammatory immune response and reduce systemic bacterial burden during sepsis.

#### 2.2.2. Series 2: Effect of Ed-LPMs in Meropenem Treated Fecal Sepsis

After establishing that Ed-LPM could effectively modulate the immune response to sepsis whilst reducing bacterial burden, we sought to determine if Ed-LPM was also effective in a combination therapy using the standard of care antibiotic Meropenem.

**Figure 3:**
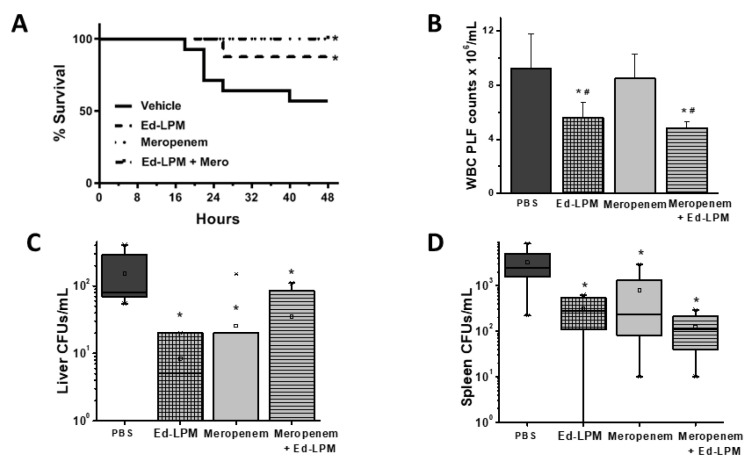
Effect of Ed-LPMs in antibiotic treated fecal sepsis. Mortality was significantly increased with PBS (6/14, 43%) compared to Ed-LPM therapy (1/8, 12%), Meropenem therapy (0/8, 100%), or combined Meropenem and Ed-LPM therapy (0/8, 100%) (Panel **A**, *p* = 0.021). Ed-LPM therapy—both alone and combined with Meropenem therapy—decreased peritoneal white blood cell counts (Panel **B**, *p* = 0.023); in contrast, Meropenem alone had no effect. Ed-LPM therapy, Meropenem, and the combination therapy each reduced bacterial burden in the liver (Panel **C**, *p* = 0.011) and spleen (Panel **D**, *p* < 0.001) compared with PBS-treated septic group. Note: Different batch of cecal slurry was used in this series; *n* = 8/group except for ED-LPM = 7; * *p* < 0.05 vs. Sepsis + PBS group, ^#^ *p* < 0.05 vs. Meropenem + PBS group’ ‘x’ in panel C is an outlier value.

Thirty-eight (38) animals were entered into this study, with 14 randomized to receive vehicle, 8 to Ed-LPM therapy, 8 to Meropenem therapy, and 8 to Meropenem plus Ed-LPM therapy. Mortality was significantly increased in septic animals that received PBS (6/14, 43%) compared to Ed-LPM therapy (1/8, 12.5%), Meropenem therapy (0/8, 100%), or combined Meropenem and Ed-LPM therapy (0/8, 100%) (Figure 3A, *p* = 0.021). From these animals, we assessed the inflammatory cells, cytokines, and systemic bacterial burden.

##### Inflammatory Cells and Cytokines

Ed-LPM therapy—both alone and combined with Meropenem—decreased peritoneal white blood cell counts (values in 10^6^/mL for Sepsis + PBS = 9.2 +/− 2.5 vs. Ed-LPM = 5.7 +/− 1.1, Mero + PBS = 8.5 +/− 1.8 vs. Mero + Ed-LPM = 4.90 +/− 0.52; *p* = 0.001) (Figure 3B); conversely, Meropenem alone had no effect on peritoneal inflammatory cell infiltration (Figure 3B and Figure S2). Ed-LPM therapy alone or with Meropenem decreased peritoneal lavage concentrations of TNFα (Figure S1C, *p* < 0.001), and increased that of IL-10 (Figure S1D, *p* = 0.049), compared to vehicle threated septic animals.

In comparison to Meropenem therapy, Ed-LPM more effectively modulated the immune response, increasing peritoneal IL-10 concentrations (Figure S1D), reducing peritoneal neutrophil counts (Figure S2A), favorably modulating the peritoneal macrophage to neutrophil ratio (Figure S2B,C) in these septic animals.

##### Bacterial Burden

The systemic bacterial CFUs were also measured in the liver and spleen. Animals receiving Ed-LPM therapy, Meropenem, and the combination therapy each displayed reduced bacterial burden in the liver (values in 10^2^ CFU/mL for Sepsis + PBS = 1.66 +/− 1.3 vs. Ed-LPM 0.3 +/− 0.6, Mero + PBS vs. 0.26 +/− 0.55, Mero + Ed-LPM 0.35 +/− 0.5, *p* = 0.011) (Figure 3C) and spleen (values in 10^3^ CFU/mL for Sepsis + PBS = 3.26 +/− 2.73 vs. Ed-LPM 0.28 +/− 0.25, Mero + PBS 0.78 +/− 1.2 vs. Mero + Ed-LPM 0.12 +/− 0.1, *p* = 0.001) (Figure 3D) compared with the PBS-treated septic group.

Taken together, these data suggest that Ed-LPM can both reduce inflammation and bacterial alone and in combination with standard of care antibiotics, such as Meropenem.

### 2.3. Ex Vivo/In Vitro Experiments

Following our observations that Ed-LPM contributed to bacterial load and inflammatory reductions in septic animals, we sought to understand the mechanisms by which this may occur. We performed several functional ex vivo and in vitro experiments with peritoneal macrophage and neutrophil cells obtained from the experimental animals of series 1 and 2.

#### 2.3.1. Peritoneal Macrophages

**Figure 4:**
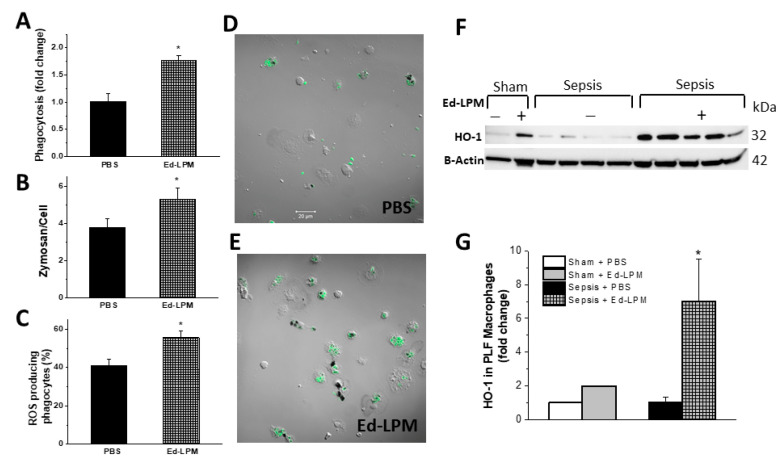
Ed-LPM treatment of septic rats enhances peritoneal macrophage function. Macrophages isolated from the peritoneum of Ed-LPM treated animals 48 h after sepsis induction demonstrated and more effective serum opsonized zymosan (SOZ) phagocytosis (Panel **A**,**B**) and higher ROS production (dark formazan spots) (Panel **C**). Representative images of peritoneal macrophage SOZ phagocytosis and ROS production are presented in (Panels **D**,**E**). Quantification of 3 experiments is shown with samples done in duplicate; * *p* < 0.05 vs. PBS group. *n* = 4–5 rats/group; *p* < 0.0001 for A & C and *p* = 0.001 for B. Peritoneal macrophages isolated from septic animals treated with Ed-LPMs express more HO-1 compared to macrophages from vehicle-treated septic rats (Panel **F**,**G**). * *p* < 0.05 vs. Sepsis + PBS group (*t*-test, *p* < 0.0001); *n* = 8 for PBS-treated and *n* = 10 for Ed-Mf treated septic group. Sham groups have *n* = 2/group (for comparison purposes only).

Macrophages isolated from the peritoneum of Ed-LPM treated animals 48 h after sepsis induction demonstrated increased serum opsonized zymosan (SOZ) phagocytosis (PBS = 1 +/− 0.13 vs. Ed-LPM 1.77 +/− 0.079, *p* < 0.0001) (Figure 4A,B), and higher ROS production (dark formazan spots) (PBS 41 +/− 4.1 vs. Ed-LPM 55.05 +/− 3.2, *p* < 0.0001) (Figure 4C). This was determined using fluorescence microscopy as exemplified in images of peritoneal macrophage SOZ phagocytosis and ROS production presented in Figure 4D,E. Additionally, Western blot analysis revealed that peritoneal macrophages isolated from septic rats treated with Ed-LPMs expressed more HO-1 compared to macrophages from vehicle-treated septic rats (Figure 4F,G).

We further confirmed that the Ed-LPMs were functionally active in vitro, as demonstrated by their ability to phagocytose SOZ (green) particles and to produce ROS (dark spots) (Figure S3A) and retained the label for 48 h (Figure S3B). We then determined that approximately 12% of the macrophages recovered from the peritoneal cavity in vivo 48 h post sepsis induction were labeled with red tracker dye, indicating that they were Ed-LPMs (Figure S3C,D).

#### 2.3.2. Peritoneal Neutrophils

**Figure 5:**
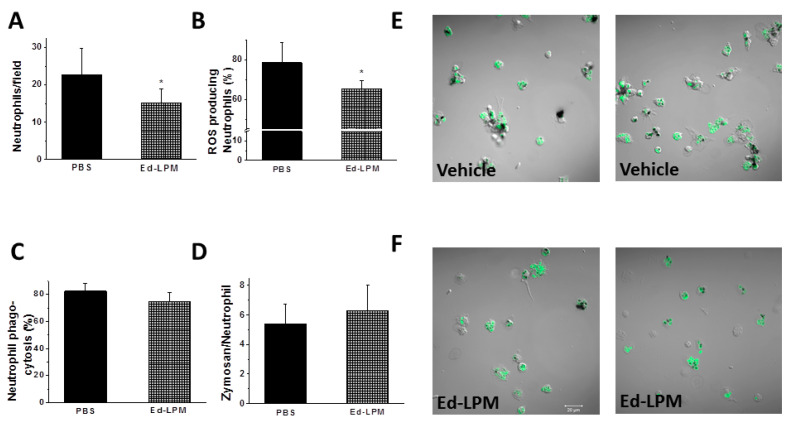
Ed-LPM therapy attenuated peritoneal neutrophils in septic rats. Ed-LPM therapy decreased neutrophil activation, as demonstrated by a lower number of neutrophils attached on the cover slip and counted per field (Panel **A**, *p* = 0.042), and decreased neutrophil ROS production (Panel **B**, *p* = 0.014), but did not alter neutrophil phagocytosis (Panels **C**,**D**). Representative images of peritoneal phagocytosis and ROS production are provided (Panels **E**,**F**). Quantification from 5 images from 3 experiments done in duplicate; * *p* < 0.05 vs. PBS group. *n* = 4–5 rats/group; *p* < 0.042 for Panel **A** and *p* = 0.014 for **B**.

Ed-LPM therapy decreased the number of activated peritoneal neutrophils (Figure 5A), and decreased neutrophil ROS production (Figure 5B), but did not alter neutrophil phagocytosis (Figure 5C,D). Representative images of peritoneal phagocytosis and ROS production are shown in Figure 5E,F. Neutrophils are often a primary immune cell targeted to sites of infection and inflammation but they are transient and die quickly after performing their functional duties (e.g., phagocytosis). Macrophages are often responsible for clearing these dead neutrophils to accelerate infection and inflammation resolution in a process called efferocytosis. Ed-LPM therapy increased macrophage efferocytosis of peritoneal neutrophils in septic rats. Representative images of efferocytosis demonstrate more effective macrophage efferocytosis of neutrophils in Ed-LPM treated septic rats (Figure S4A) compared with PBS-treated rats (Figure S4B). This effect was more marked at 72 h than at 48 h post-sepsis induction (Figure S4C). We then considered that Ed-LPM mediated neutrophil efferocytosis is a result of increased peritoneal macrophage activation of Axl (i.e., phospho-Axl) receptors.

Western blot analysis confirmed that Ed-LPM treated septic rats displayed increased pAxl in their PLF macrophages in comparison to the controls (Figure S5). Ed-LPM therapy also increased peritoneal neutrophil apoptosis as evidenced by increased apoptotic markers (Casp-3 and Bax) at 72 h following sepsis induction (Figure S6A–C). Ed-LPM treated septic animals demonstrated increased percentages and numbers of peritoneal macrophages and decreased neutrophil proportions and absolute number in septic rats (Figure S6D,E).

#### 2.3.3. Meropenem and Ed-LPM Function

Meropenem did not modulate the function of rat Ed-LPMs (Figure S7). There was no difference in the capabilities of peritoneal macrophages to phagocytose SOZ particles or to produce ROS following Meropenem exposure.

Taken together, these data suggest that Ed-LPMs help to direct the inflammatory immune response in peritoneal sepsis in favour of clearing dead and dying neutrophils by efferocytosis that is mediated by Axl. This augmented phagocytic capacity of Ed-LPMs further suggests the therapeutic merit of Ed-LPMs to address and control infection and inflammation during sepsis.

## 3. Discussion

In the present study, we demonstrate that embryonic-derived large peritoneal-like macrophages (Ed-LPMs) are able to control the severity of peritoneal fecal sepsis. We show that in a model of fecal sepsis in rats, the animals receiving Ed-LPMs exhibited attenuated leukocyte infiltration—especially neutrophils—to the peritoneal cavity and lungs. Additionally, a decrease in the inflammatory cytokine TNFα and an increase in the anti-inflammatory cytokine IL-10 content was found in the peritoneal lavage fluid of Ed-LPM treated rats. The bacterial burden in multiple organs—including the lungs, liver, and spleen—was also reduced in septic animals that were treated with Ed-LPMs. These data suggest that Ed-LPM therapy has the potential to control bacterial sepsis.

Peritoneal macrophages (PM) play pivotal roles in the innate immune response to fecal peritonitis induced sepsis [15,20] and, thus, represent attractive therapeutic targets. Two subsets of PMs co-exist in the peritoneal cavity that has been classified according to their morphology, namely large PMs (LPMs) and small PMs (SPMs). LPMs originate from the fetal yolk sac [15,21] and are tissue macrophages [22]. LPMs are crucial for maintenance of tissue homeostasis and repair [15,23]. In contrast, SPMs originate from bone-marrow-derived myeloid precursors (e.g., monocytes) and constitute a minor subset in the healthy peritoneal cavity [15]. In the presence of peritoneal infectious or inflammatory stimuli, the peritoneal macrophage sub-population composition is dramatically altered, where LPMs become nearly undetectable against a large influx of monocyte-derived SPMs [15]. Though SPMs represent a minor population during non-inflammatory states, their rapid increase during inflammation can be associated with significantly increased systemic cytokine levels, which can often cause collateral tissue and organ damage [12,15]. Changing the macrophage balance by increasing the LPM numbers could therefore reduce some of the collateral damage sustained by tissue and organs and additionally support the reduction in bacterial proliferation. Thus, we considered that introduction of exogenous LPM-like cells could change this macrophage balance by increasing the proportion of functional LPMs. In this study, we show that therapeutic addition of embryonic-derived LPMs into an animal model of fecal sepsis attenuates sepsis-induced systemic injury, reduces inflammatory mediators, increases anti-inflammatory mediators, and decreases bacterial loads.

We used pluripotent embryonic rat stem cells to develop embryonic-derived large peritoneal-like macrophages (Ed-LPMs). These cells share phenotypic characteristics of natural LPMs including expression of surface markers SIRP-alpha, CD11b/c, TLR4, and CD80 but not CD62L, a well-known SPM marker [15]. Similar to our published method of generating alveolar-like macrophages [15], we pre-adapted the Ed-LPMs in vitro with GM-CSF and M-CSF, albeit with lower concentrations of GM-CSF than used previously for airway macrophages to replicate closer the peritoneal GM-CSF environment [24]. To our knowledge, this is the first report of primitive embryonic-derived macrophages adapted to the peritoneum to be used for therapeutic attenuation of fecal peritoneal sepsis.

The standard of care for most bacterial infections is antibiotic therapy. However, in recent years, alternative and complimentary cellular therapies have been considered including mesenchymal stromal cell (MSC) therapy. Reports suggest that MSC therapy can address some aspects of sepsis in animal models, though mechanisms of action still remain unclear [25]. Major criticisms of this approach include lack of direct functional immunological activity on the target bacteria and a rapid dissipation of MSCs within target tissues or organs. Macrophages, conversely, show direct functional immune effects including direct bactericidal and efferocytotic clearance effects. Here, we found that the magnitude of the effect of Ed-LPMs on animal survival and bacterial loads was similar in magnitude to the administration of high dose Meropenem, a broad-spectrum antibiotic with proven efficacy in abdominal sepsis [26] and in the critically ill [27]. We also show the effectiveness of Ed-LPM in the absence of antibiotic treatment. Additionally, we show data supporting direct interaction of Ed-LPMs with bacteria and describe clear mechanistic features of these phenomena. We observed an improvement in inflammatory markers, such as reduction in TNFα and increase in IL-10 in PLF with Ed-LPM in combination with Meropenem; however, inflammatory cell infiltrates were decreased in the Ed-LPM group whereas the Meropenem alone did not contribute to this phenomenon. When compared to Meropenem therapy, Ed-LPM therapy more effectively modulated the immune response, reducing peritoneal cavity neutrophil counts, favorably modulating the peritoneal macrophage to neutrophil ratio, enhancing intra-peritoneal macrophage phagocytosis, and increasing IL-10 concentrations, in these septic animals. These immunomodulatory effects raise the possibility that that Ed-LPM therapy could be effective irrespective of the antibiotic resistance patterns of the pathogens, an important advantage in the era of multi-drug antibiotic resistant pathogens.

The mechanisms underlying these effects appeared in part due to enhanced peritoneal macrophage bacterial phagocytosis and killing, as evidenced by the fact that macrophages retrieved from the peritoneal cavity of Ed-LPM treated animals demonstrated enhanced phagocytosis of bacterial products, and enhanced macrophage phagosomal ROS production, which is essential for killing of phagocytosed bacteria. Ed-LPM therapy enhanced peritoneal macrophage HO-1 concentrations, a mechanism of action we have previously demonstrated to be important in enhancing macrophage function [28].

A potentially important and novel mechanism of action of Ed-LPMs appears to be mediated via modulation of the function and lifespan of infiltrated neutrophils from the peritoneal cavity. In animals treated with Ed-LPMs, peritoneal neutrophil counts were reduced at 48 and 72 h following induction of abdominal sepsis. In addition, Ed-LPM therapy decreased peritoneal neutrophil ROS production. During tissue inflammation and injury, such as occurs during bacterial infection, infiltrating neutrophils may cause host tissue damage via uncontrolled ROS production and release [29]. In fact, targeting neutrophil ROS production has been identified as a therapeutic target for chronic inflammatory disorders such as inflammatory bowel disease [30], and acute conditions such as acute lung injury [31], in addition to sepsis [29]. Ed-LPM therapy also enhanced bacterial clearance from the peritoneal cavity by enhancing neutrophil apoptosis, and increasing macrophage efferocytosis of infiltrated neutrophils. We further evaluated the functional attributes of the peritoneal macrophages from the peritoneal lavage cells and determined that in the Ed-LPM treated group, efferocytosis—the phagocytic engulfment of apoptotic cells—occurred at an increased efficiency, especially in targeting apoptotic neutrophils. This may explain why we observed significantly fewer neutrophils in the Ed-LPM treated group, while neutrophil apoptosis was increased. These data corresponded to increased activation of Axl receptors in PLF macrophages supporting the concept that Ed-LPM therapy promotes cellular mechanics involving phagocytosis. Moreover, these observations support the well-documented phenomenon that macrophage phagocytosis of apoptotic cells suppresses inflammatory cytokine production and promotes the secretion of anti-inflammatory cytokines such as IL-10 [32,33]. These observations further support the contention that Ed-LPMs directly enhance the clearance of injurious material in the peritoneum while also indirectly enhancing the functional attributes of resident peritoneal macrophages. It is worth noting that a more robust initial innate response to clear the injury appears achievable with Ed-LPM therapy and this could reduce the number of cells and duration for which the highly inflammatory monocyte-derived SPMs infiltrate the site of injury. Thus, it would appear that Ed-LPM therapy could mitigate and control the SPM-mediated immune response that can be highly injurious to the host as a result of the recruitment signals required for such an intense inflammation.

There are, however, important limitations to the current data. First, these studies were carried out in a rodent model, albeit a highly clinically relevant model of sepsis, and so caution must be exercised in clinical extrapolation, as these models cannot fully replicate the complexities of human sepsis. Second, these Ed-LPMs are of rodent origin, and studies using human-derived “peritoneal-like” LPMs are required prior to any consideration of clinical translation of these findings.

Nevertheless, our observations of Ed-LPM therapy during experimental fecal sepsis show effective reductions in bacterial load from multiple organs and enhancements in the clearance of bacteria and apoptotic neutrophils whilst also suppressing inflammation and promoting resolution of injury. This is consistent with previous work we have published using embryonic-derived alveolar-like macrophages to resolve pulmonary injury [17]. These findings suggest that macrophage transplantation therapy may ultimately have potential as a testable therapeutic strategy for sepsis and justify further investigation into the use of macrophages as potential therapeutics for infection and inflammation related diseases.

## 4. Materials and Methods

All work was approved by the Animal Care and Use Committee of the Keenan Research Centre for Biomedical Science of St. Michael’s Hospital—Unity Health Toronto, Toronto (ACC648), and conducted under license from Health Canada. All studies on human peripheral blood mononuclear cells were approved by the Research Ethics Board of St. Michael’s Hospital—Unity Health Toronto, Toronto (REB: 14-278). All experiments were carried out in the research laboratories at St. Michael’s Hospital—Unity Health Toronto, Toronto. More detailed methods are available in the supplemental data file.

### 4.1. Rat Embryonic-Derived Large “Peritoneal-Like” Macrophages (Ed-LPM)

Directed differentiation of rat pluripotent stem cells (PSCs) was used to produce expandable Ed-LPMs using the protocol outlined by Litvack and colleagues [17]. Briefly, rat embryonic stem cells were maintained at pluripotency in 2*i* + LIF serum-free stem cell media as previously described [17,34]. The ESCs were removed from the 2*i* pluripotency media and then cultured in mesoderm-inducing media, followed by hematopoiesis inducing media. The primitive macrophages budding off cell clusters were selected based on attachment to ultra-low adhesion plates (ULA plates—Corning 3471) over the course of a 21–28 day culture and expansion period. These PSC-derived primitive macrophages were conditioned to a “LPM-peritoneal-like” phenotype with 2 ng/mL granulocyte–macrophage colony–stimulating factor (GM-CSF) and 10 ng/mL macrophage colony-stimulating factor (M-CSF) (Thermo Fisher Sci, Burlington, ON, Canada). Flow cytometry was completed as previously described [17]. Briefly, cells were harvested from adherent macrophage cultures using TrypLE (Life Technologies, Burlington, ON, Canada) cell dissociation reagent. Cells were coated with an anti-rat Fc-block in sorting buffer (Hank's Balanced Salt Solution - HBSS with 2% serum and 1% HEPES) and then stained with the desired primary fluorescently-labelled antibody or antibodies at the indicated dilution (see Supplemental Table S1). Fluorescent cell populations were acquired and analyzed on the Becton Dickenson Gallios 10/3 bench top flow cytometer and analyzed using the Kaluza flow cytometry software suite (Becton Dickenson). Analytic gating strategies were devised based on live cells as determined by forward scatter and side scatter gated plots and the unstained negative controls per the cytometers’ manufacturer’s instructions (Figure S8). These cells were confirmed to be *Myb*^−^ (Figure S9) as has been previously described [17]. Cultured cells were washed and resuspended in phosphate buffered saline (PBS) before administration.

### 4.2. Rodent Fecal Sepsis Protocol

Specific-pathogen-free adult male Sprague Dawley rats (Charles River Laboratories, Saint Constant, QC, Canada; 350–450 g) were used in all experiments.

#### 4.2.1. Cecal Slurry Stock Preparation

For each experimental series, a cecal slurry batch (Batch A and B) was prepared. For each batch, 20 rats were euthanized, the cecum dissected, and the cecal contents combined, mixed with sterile water and filtered through sterile meshes (first 380 µm; then 190 µm), and added to an equal volume of 30% glycerol in PBS [35]. The stock was aliquoted into 5 mL cryovials, frozen and stored at −80 °C.

#### 4.2.2. Fecal Slurry Sepsis Induction

Rats were anesthetized with isoflurane and 2 mL of blood was drawn from the tail vein, mixed with 2 mL of cecal slurry (0.5 g/kg), divided in two equal parts and allowed to clot for 15 min before instillation. The rat abdomen was shaved, cleaned (with isopropyl alcohol and betadine solution). The two clots were instilled into peritoneal cavity through a 1 cm midline incision, one on each side. The muscle layer and the skin were sutured in a continuous and discontinuous pattern, respectively. The skin was cleaned with hydrogen peroxide. Slow-release buprenorphine HCl, 1 mg/kg (Chiron Compounding Pharmacy, Guelph, ON, Canada), was injected subcutaneously before surgery and lactated Ringer’s solution (20 mL/kg) was administered at surgery and every 12 h until harvest.

Preliminary experiments determined the cecal slurry dose required to produce sepsis over a 48–72 h period. In series 1, the batch A of cecal slurry was used and this produced a low mortality rate (~15%) in vehicle-treated animals. For series 3, we used batch B of cecal slurry, which produced a mortality rate in vehicle-treated animals ~43%. Subsequently, taxonomy data (Figure S10) demonstrated potentially relevant differences in composition of these two batches of cecal slurry that could account for different survival and outcome of sepsis.

#### 4.2.3. Experimental Design

In Series 1, the safety and efficacy of Ed-LPM in fecal sepsis was evaluated. Four hours following induction of fecal sepsis or sham procedure, animals were randomized to receive intraperitoneal administration of Ed-LPMs (10 million/kg) or vehicle (PBS), in a 4 group design. Series 2 evaluated the efficacy of Ed-PM therapy (10 million/kg) in the presence of meropenem antibiotic therapy (Fresenius Kabi Canada Ltd., Toronto, ON, Canada) given (25 mg/mL, i.v.) at 4, 18, and 30 h after sepsis induction, in a 4 group design.

In each series as appropriate, the vehicle (PBS) or Ed-LPM (at the dose of 10 mill/kg) were injected 4 h after sepsis induction IP through a 20 G × 48 mm catheter inserted in place and sutured to the skin at the time of surgery and removed after injection.

#### 4.2.4. Assessment of Septic Injury

At 48 h or 72 h after sepsis induction and cell/antibiotic treatments, all animals were re-anesthetized, hemodynamic and oxygenation indices assessed [36,37,38,39], and the animals were subsequently euthanized by exsanguination.

### 4.3. Ex Vivo Analyses

Differential leukocyte counts were measured in peritoneal lavage fluid (PLF) and bronchoalveolar lavage (BAL) fluid.

Phagocytosis and superoxide production was assessed in peritoneal macrophages and neutrophils isolated from treated or non-treated rats (receiving vehicle) by Ficoll gradient and seeded on cover slips of 12-well plate. Macrophages and activated neutrophils attach to the plate. Phagocytic capacity was assessed using Alexa-488-conjugated serum opsonized zymosan, and enzyme-labelled *E. coli* particles, while reactive oxygen species (ROS) production was determined using the Zymosan/Nitroblue tetrazolium (NBT) assay [40,41].

Macrophage efferocytosis was assessed in peritoneal macrophages and neutrophils isolated from rats 48 h and 72 h after sepsis induction. Macrophages were seeded on cover slips of 12-well plate and allowed 1 h to attach. Neutrophils were labelled blue using Hoechst dye and neutrophils were incubated with adhered macrophage in 5:1 ratio. The cells were then incubated at 37 °C for 30 min. Non-phagocytosed neutrophils were removed by three washes with PBS and the cells were fixed in 4% paraformaldehyde in PBS for 15 min. Cells were visualized by confocal microscopy using laser scanning Zeiss LSM700 microscope equipped with a single pinhole (Carl Zeiss Microscopy GmbH, Peabody, MA, USA) and ZEN software (2012 blue edition). Counting of macrophages that engulfed (blue) neutrophils was done in 8–10 randomly chosen fields/slide using Image-J (1.48a, NIH, USA) software and efferocytosis was calculated as percentage of macrophage that phagocytosed neutrophils over total number of macrophages.

ED-LPMs were labeled with red tracker dye (C34552, Molecular probes, Life Technologies, Burlington, ON, Canada) 2000× dilution for 30 min prior to administering Ed-LPM to the rats or for in vitro experimentation. Preliminary in vitro experiments showed that such labeling did not alter Ed-LPM functionality. Labeled Ed-LPM were then injected intra-peritoneally into the septic animals and the recovery of labeled Ed-LPMs was assessed after 48 h, and their functionality after recovery was compared to non-labeled PMs. Macrophages and neutrophils were also lysed to perform Western Blot (WB) analysis for apoptotic markers (Caspase3 & Bax) in neutrophils and scavenger receptor expression and activity in macrophages (Axl).

Western blot procedure: Western blot analysis was performed according to an established protocol [42]. Briefly, tissues were homogenized in TNE buffer (0.05 M Tris/HCl, pH7.4, 0.1 M NaCl, 1 mM EDTA) supplemented with 1% Triton X-100 and protease/phosphatase inhibitors and equal protein amounts were fractionated on 4–12% gradient NuPAGE gels (Invitrogen, Burlington, ON, Canada) and transferred to a polyvinylidene difluoride (PVDF) membrane (Immobilon-P, Millipore Corp, Bedford, MA, USA). After blocking with 5% milk in Tris-Buffered Saline and Tween 20 (TBS-T), the blot was incubated with primary antibody for 2 h or overnight, followed by a secondary antibody conjugated with horseradish peroxidase for 1 h. The following primary antibodies were used: Caspase-3 (total and cleaved) and Bax, both from Cell Signaling Technologies (Danvers, MA, USA), used at 1:1000 dilution; Phospho-Axl (Y779: R&D Systems cat no. AF2228), used at 1 ug/mL, and total Axl (Proteintech Group cat no. 13196-1-AP), used at 1:1000 dilution. Signals were detected using an ECL-Plus kit (Amersham Biosci, Piscataway, NJ, USA). Band intensities were quantified and expressed relative to that of β-actin, 1:10,000 (mouse IgG1, Sigma-Aldrich, Oakville, ON, Canada).

### 4.4. Statistical Analysis

Data are presented as Mean +/− SD and were analyzed using GraphPad Prism (GraphPad^®^ software, La Jolla, CA, USA). The distribution of all data was tested for normality using Kolmogorov-Smirnov tests. Data were analyzed by one-way ANOVA, or ANOVA on Ranks with post hoc testing using the Newmann–Keuls Multiple Comparison Test or Dunnet’s tests, as appropriate. In series 2, which examined survival, the Log Rank test was used. Underlying model assumptions were deemed appropriate on the basis of suitable residual plots. A two-tailed *p* value of <0.05 was considered significant.

## Data Availability

The data presented in this study are available on request from the corresponding author. Key data are stated in the text.

## Supplementary Materials

The following are available online at https://www.mdpi.com/1422-0067/22/6/3190/s1.
