# Supplementary material for: Embryonic-Derived Myb− Macrophages Enhance Bacterial Clearance and Improve Survival in Rat Sepsis
Source: Int J Mol Sci. 2021 Mar 20;22(6):3190. doi: 10.3390/ijms22063190 (PMC8004006; doi:10.3390/ijms22063190)
Supplement: Supplementary file 1 [file ijms-22-03190-s001.pdf]

ONLINE DATA SUPPLEMENT

Table of contents:

EXPANDED MATERIALS AND METHODS 2
Supplemental Tables 9
Supplemental Figures 9
References 18

EXPANDED MATERIALS AND METHODS

All work was approved by the Animal Care and Use Committee of the Keenan Research Centre for Biomedical Science of St Michael's Hospital, Toronto (ACC648), and conducted under license from Health Canada. All studies on human peripheral blood mononuclear cells were approved by the Research Ethics Board of St Michael's Hospital, Toronto (REB: 14-278). All experiments were carried out in the research laboratories at St Michael's Hospital, Toronto. More detailed methods are available in the online data supplement.

Rat Embryonic-derived Large 'Peritoneal-like' Macrophages (Ed-LPM)

Directed differentiation of rat pluripotent stem cells (PSCs) was used to produce expandable Ed-LPMs. PSC-derived macrophages were conditioned to a 'LPM-peritoneal-like' phenotype with 2 ng/mL granulocyte–macrophage colony–stimulating factor (GM-CSF) and 10ng/mL macrophage colony–stimulating factor (M-CSF) [Thermo Fisher Sci, Burlington, ON, Canada]. Flow cytometry was completed as previously described [1]. Briefly, cells were harvested from adherent macrophage cultures using TrypLE (Life Technologies) cell dissociation reagent. Cells were coated with an anti-rat Fc-block in sorting buffer (HBSS with 2% serum and 1% HEPES) and then stained with the desired antibody or antibodies at the indicated dilution (see Table 1). Fluorescent cell populations were acquired and counted on the Becton Dickenson Gallios 10/3 bench top flow cytometer and

analyzed using the Kaluza flow cytometry software suite (Becton Dickenson). Analytic gating strategies were devised based on the unstained negative controls per the cytometers' manufacturer's instructions. Cultured cells were washed and resuspended in phosphate buffered saline (PBS) before administration.

Rodent Fecal Sepsis Protocol

Specific-pathogen-free adult male Sprague Dawley rats (Charles River Laboratories, Quebec, Canada; 350-450 g) were used in all experiments.

Cecal slurry stock preparation: For each experimental series, a cecal slurry batch (Batch A and B) was prepared. For each batch, 20 rats were euthanized, the cecum dissected, and the cecal contents combined, mixed with sterile water and filtered through sterile meshes (first 380 μm; then 190 μM), and added to an equal volume of 30% glycerol in PBS [2]. The stock was aliquoted into 5 mL cryovials, frozen and stored at -80°C.

Fecal slurry sepsis induction: Rats were anesthetized with isoflurane and 2 mL of blood was drawn from the tail vein, mixed with 2 mL of cecal slurry (0.5 g/kg), divided in two equal parts and allowed to clot for 15 min before instillation. The rat abdomen was shaved, cleaned (with isopropyl alcohol and betadine solution). The two clots were instilled into peritoneal cavity through a 1 cm midline incision, one on each side. The muscle layer and the skin were sutured in a continuous and discontinuous pattern, respectively. The skin was cleaned with hydrogen peroxide.

Slow release buprenorphine HCl, 1mg/kg (Chiron Compounding Pharmacy, Guelph, Canada) was injected subcutaneously before surgery and lactated Ringer's solution (20mL/kg) was administered at surgery and every 12hrs until harvest.

Preliminary experiments determined the cecal slurry dose required to produce sepsis over a 48-72 hour period. In series 1 the batch A of cecal slurry was used and this produced a low mortality rate (~15%) in vehicle-treated animals. For series 3 we used batch B of cecal slurry, which produced a mortality rate in vehicle-treated animals ~43%. Subsequently, taxonomy data (**Figure S8**) demonstrated potentially relevant differences in composition of these two batches of cecal slurry that could account for different survival and outcome of sepsis.

Experimental Design: *In* ***Series 1***, the safety and efficacy of Ed-LPM in fecal sepsis was evaluated. Four hours following induction of fecal sepsis or sham procedure, animals were randomized to receive intraperitoneal administration of Ed-LPMs (10 million/kg) or vehicle (PBS), in a 4 group design. ***Series 2*** evaluated the efficacy of Ed-PM therapy (10 million/kg) in the presence of meropenem antibiotic therapy (Fresenius Kabi Canada Ltd., Toronto, ON, Canada) given (25 mg/mL, i.v.) at 4, 18, and 30h after sepsis induction, in a 4 group design.

In each series as appropriate, vehicle (PBS) or Ed-LPM or UC-MSC (both at the dose of 10 mill/kg) were injected 4h after sepsis induction IP through a 20Gx48mm catheter inserted in place and sutured to the skin at the time of surgery and removed after injection.

Assessment of Septic Injury: At 48 h or 72h after sepsis induction and cell/antibiotic treatments, all animals were re-anesthetized, hemodynamic and oxygenation indices assessed [3-6], and the animals were subsequently euthanized by exsanguination.

Ex vivo Analyses

Differential leukocyte counts were measured in peritoneal lavage fluid (PLF) and bronchoalveolar lavage (BAL) fluid.

Phagocytosis and superoxide production was assessed in peritoneal macrophages and neutrophils isolated from treated or non-treated rats (receiving vehicle) by Ficoll gradient and seeded on cover slips of 12-well plate. Macrophages and activated neutrophils attach to the plate. Phagocytic capacity was assessed using Alexa-488-conjugated serum opsonized zymosan, and enzyme-labelled *E. coli* particles, while reactive oxygen species (ROS) production was determined using the Zymosan/Nitroblue tetrazolium (NBT) assay [7, 8].

Macrophage efferocytosis was assessed in peritoneal macrophages and neutrophils isolated from rats 48h and 72h after sepsis induction. Macrophages were seeded on cover slips of 12-well plate and allowed 1h to attach. Neutrophils were labelled blue using Hoechst dye and neutrophils were incubated with adhered macrophage in 5:1 ratio. The cells were then incubated at 37℃ for 30 min. Non-phagocytosed neutrophils were removed by three washes with PBS and the cells were fixed in 4%

paraformaldehyde in PBS for 15 min. Cells were visualized by confocal microscopy using laser scanning Zeiss LSM700 microscope equipped with a single pinhole (Carl Zeiss Microscopy GmbH, Peabody, MA, USA) and ZEN software (2012 blue edition). Counting of macrophages that engulfed (blue) neutrophils was done in 8-10 randomly chosen fields/slide using Image-J (1.48a, NIH, USA) software and efferocytosis was calculated as percentage of macrophage that phagocytosed neutrophils over total number of macrophages.

ED-LPMs were labeled with red tracker dye (C34552, Molecular probes, Life Technologies, Burlington, ON, Canada) 2000x dilution for 30 minutes prior to administering Ed-LPM to the rats or for *in vitro* experimentation. Preliminary *in vitro* experiments showed that such labeling did not alter Ed-LPM functionality. Labeled Ed-LPM were then injected intra-peritoneally into the septic animals and the recovery of labeled Ed-LPMs was assessed after 48h, and their functionality after recovery was compared to non-labeled PMs.

Macrophages and neutrophils were also lysed to perform **Western Blot (WB) analysis** for apoptotic markers (Caspase3 & Bax) in neutrophils and scavenger receptor expression and activity in macrophages (Axl).

Western blot procedure: Western blot analysis was performed according to an established protocol (12). Briefly, tissues were homogenized in TNE buffer (0.05 M Tris/HCl, pH7.4, 0.1 M NaCl, 1 mM EDTA) supplemented with 1% Triton X-100 and protease/phosphatase inhibitors and equal protein amounts were fractionated on 4-

12% gradient NuPAGE gels (Invitrogen, Burlington, ON, Canada) and transferred to a polyvinylidene difluoride (PVDF) membrane (Immobilon-P, Millipore Corp, Bedford, MA, USA). After blocking with 5% milk in Tris-Buffered Saline and Tween 20 (TBS-T), the blot was incubated with primary antibody for 2 hours or overnight, followed by a secondary antibody conjugated with horseradish peroxidase for 1 hour. The following primary antibodies were used: Caspase-3 (total and cleaved)and Bax, both from Cell Signaling Technologies (Danvers, MA, USA), used at 1:1000 dilution; Phospho-Axl (Y779: R&D Systems cat no. AF2228), used at 1 ug/mL, and total Axl (Proteintech Group cat no. 13196-1-AP), used at 1:1000 dilution. Signals were detected using an ECL-Plus kit (Amersham Biosci, Piscataway, NJ, USA). Band intensities were quantified and expressed relative to that of β-actin, 1:10,000 (mouse IgG1, Sigma-Aldrich, Oakville, ON, Canada).

Statistical Analysis

Data are presented as Mean +/- SD and were analyzed using GraphPad Prism (GraphPad® software, La Jolla, CA, USA). The distribution of all data was tested for normality using Kolmogorov-Smirnov tests. Data were analyzed by one-way ANOVA, or ANOVA on Ranks with *post hoc* testing using Newmann-Keuls Multiple Comparison Test or Dunnet's tests as appropriate. In series 2, which examined survival, the Log Rank test was used. Underlying model assumptions were deemed

appropriate on the basis of suitable residual plots. A two-tailed *P* value of <0.05 was considered significant.

Supplemental Tables

Table S1: Surface markers used in characterization of Ed-LPMs

Marker	Company	Product number	Fluorophore	Dilution factor
**Rat Macrophage marker**	Thermo Fisher	12-0660-82	PE	1 : 100
**SIRP alpha**	Thermo Fisher	17-1720-82	APC	1 : 100
**CD11b/c**	Thermo Fisher	50-0110-80	Ef660	
**CD11b/c**	Thermo Fisher	46-0110-80	PerCP-ef710	1 : 100
**TLR4**		PA5-23125	unconjugated	1:10
**CD80**	Thermo Fisher	25-0801-80	PE-Cy7	1 : 100
**CD62L**	Thermo Fisher	50-0623-80	Ef660	1 : 100
**2° antibody**	Life Technologies	A11034	AF488	1 : 200
**Fc-block (CD32)**	BD Pharmigen	550270	unconjugated	1 : 100

Supplemental Figures

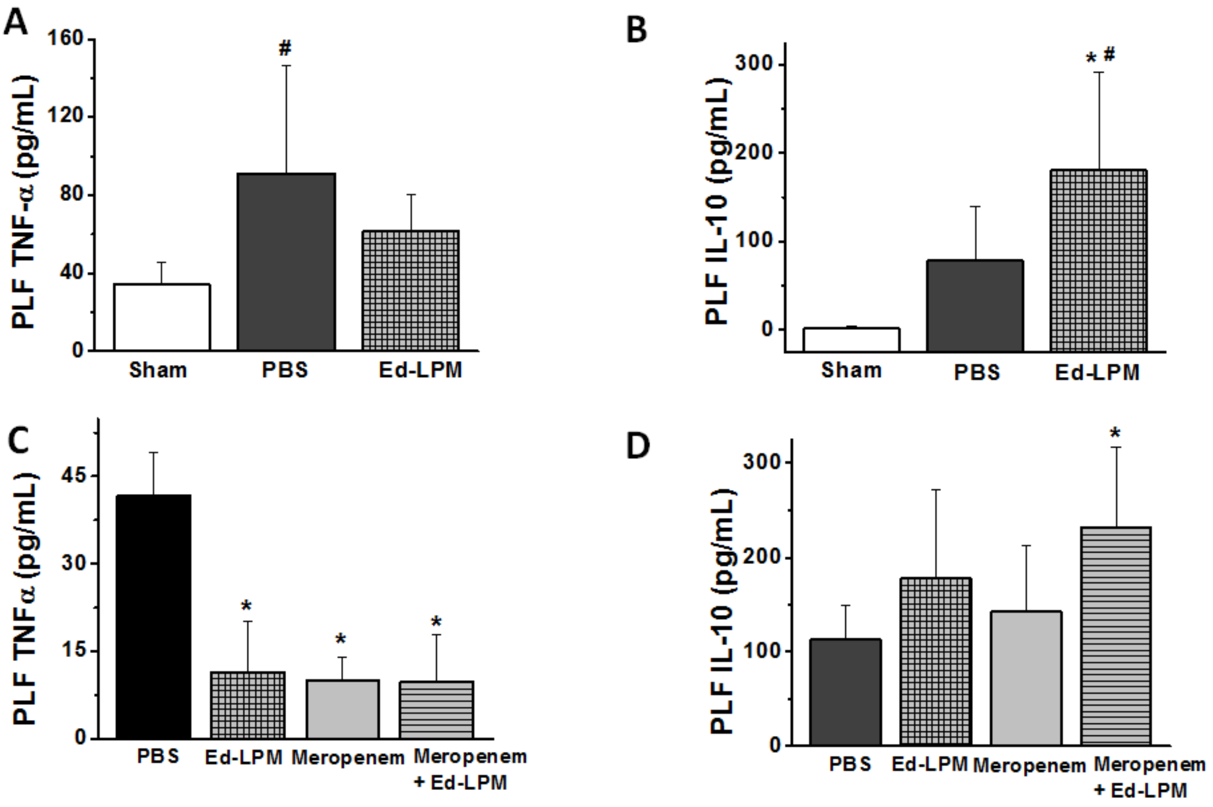

Figure S1. Ed-LPM therapy modulates the peritoneal lavage cytokine profile. Ed-LPM therapy decreased peritoneal lavage concentrations of TNFa [*Panel A*, *P*=0.018], and increased IL-10 concentrations [*Panel B*, *P*<0.001], compared to vehicle, in the septic animals. In series 2, Ed-LPM therapy also decreased peritoneal lavage concentrations of TNFa [*Panel C*, *P*<0.001], and increased IL-10 concentrations [*Panel d*, *P*=0.049], compared to vehicle or meropenem therapy, in the septic animals.

#*P* < 0.05 vs. Sham; **P* < 0.05 vs. PBS sepsis group; *N* = 6-8/group

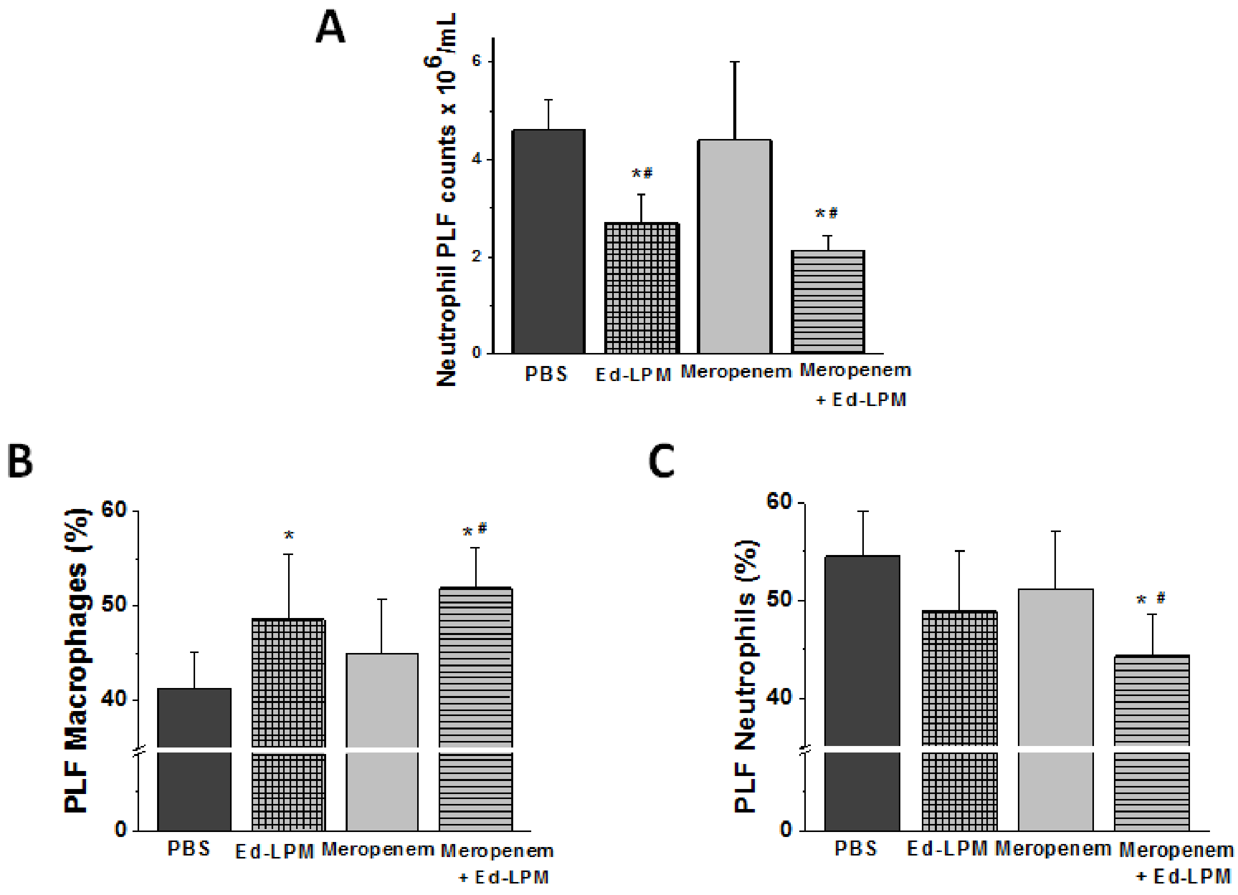

Figure S2. Meropenem did not modulate peritoneal inflammatory cell infiltrate. Meropenem treatment did not change Neutrophil abundance in the septic animals [***Panel A***, *P*=0.003]. Meropenem does not change percentage of PLF Macrophages [***Panel B***, *P*=0.004] or Neutrophils [***Panel C***, =0.006].

**P* < 0.05 vs. Vehicle (PBS) sepsis group, #*P* < 0.05 vs. Meropenem group; N=8/group except for ED-LPM=7.

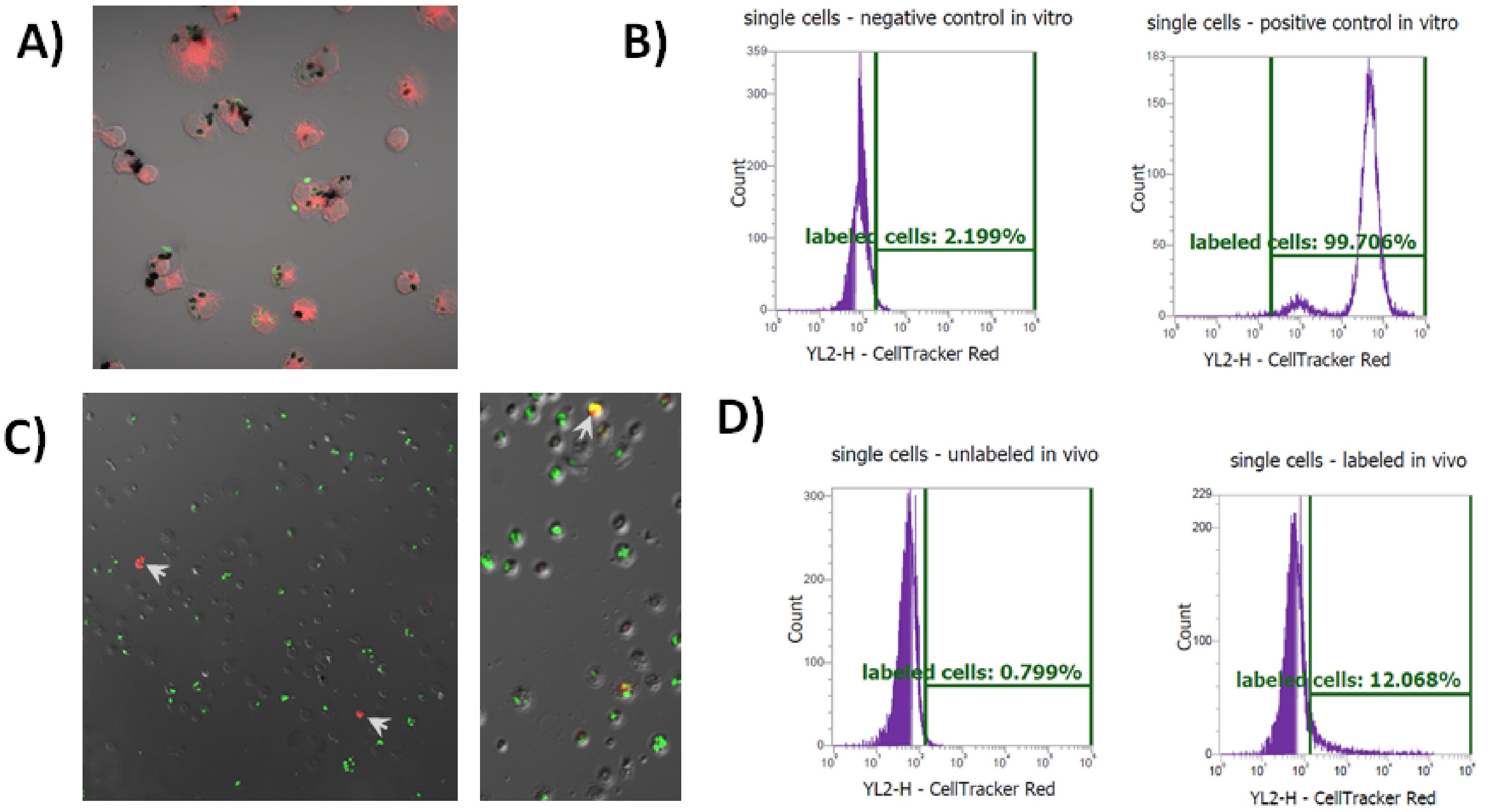

Figure S3. In vitro **labeled ED-LPM (A) demonstrate successful red-tracker uptake**.

SOZ assay done on this macrophage shows functionality of labeled Macrophage in terms of their ability to phagocytose SOZ (green) particles and ROS production (dark spots). Unlabeled and labeled macrophages are also used as negative and positive *in*

vitro control (B). *In vivo* data (C and D) are showing that ~12% of Ed-LPM labeled with red-tracker (arrows in image C) and given to the septic animals are recovered from total Macrophage isolated from PLF 48h after sepsis induction (D).

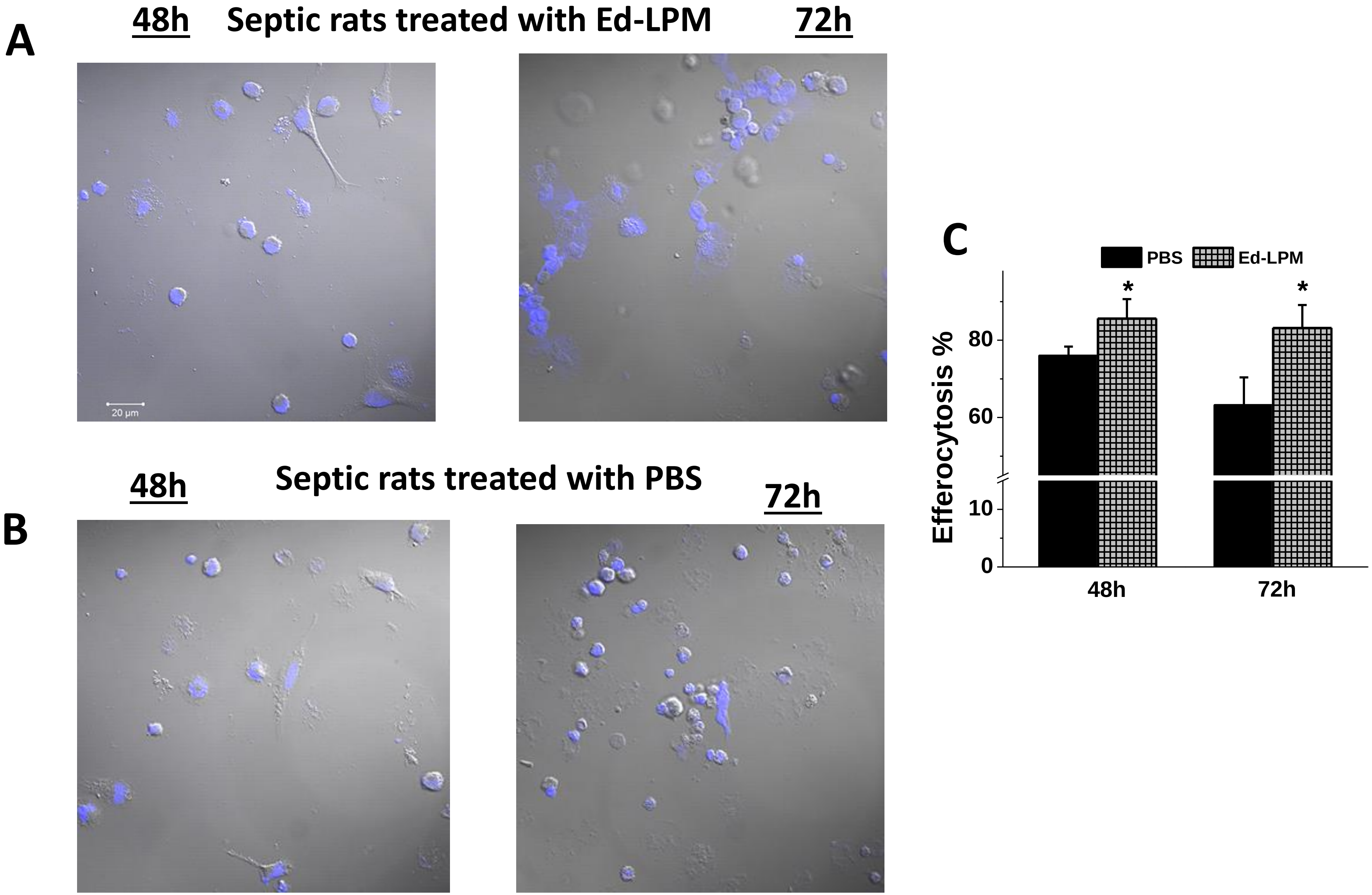

Figure S4. Ed-LPM therapy increases macrophage efferocytosis of neutrophils in septic rats

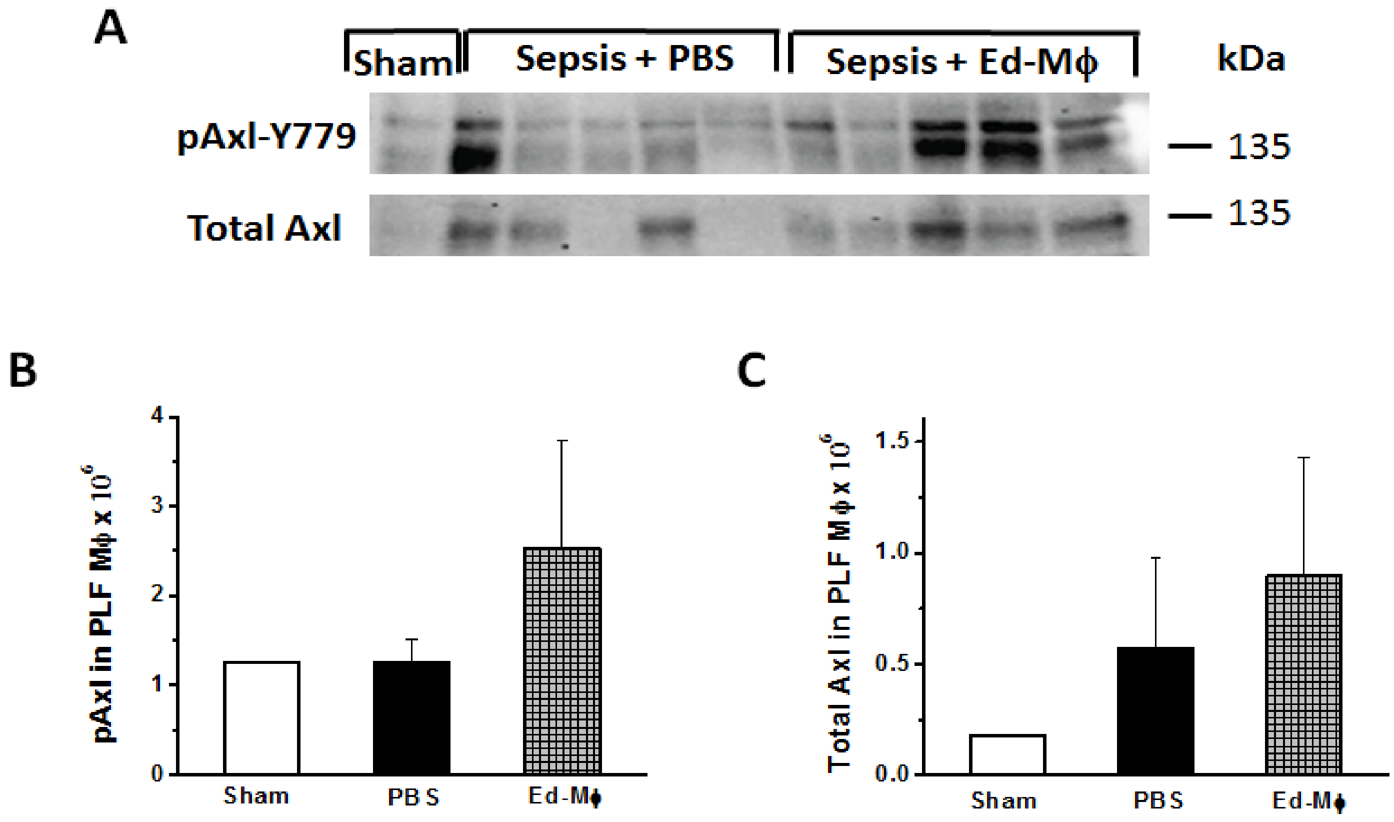

Figure S5. Ed-LPM therapy may activate peritoneal macrophage Axl receptors in septic rats. Representative images of pAxl and total Axl 48h after sepsis or Sham procedure [***Panel A***] are showing trend of increased activation of Axl (pAxl) receptors (that are involved in efferocytosis) in septic PLF Macrophage isolated from rats treated with R-Ed-LPM. Quantification is shown in ***Panels B-C***.

N = 5/group for septic rats. Sham group just for reference only. Second lane on the gel is omitted for quantification as it is not clear where pAxl band ends; *P*=0.038 one tail T-Test and *P*=0.077 two-tail T-test for pAxl [Panel B], *P*=0.15 one tail T-test and *P*=0.3 two-tail T-test for Total Axl [Panel C].

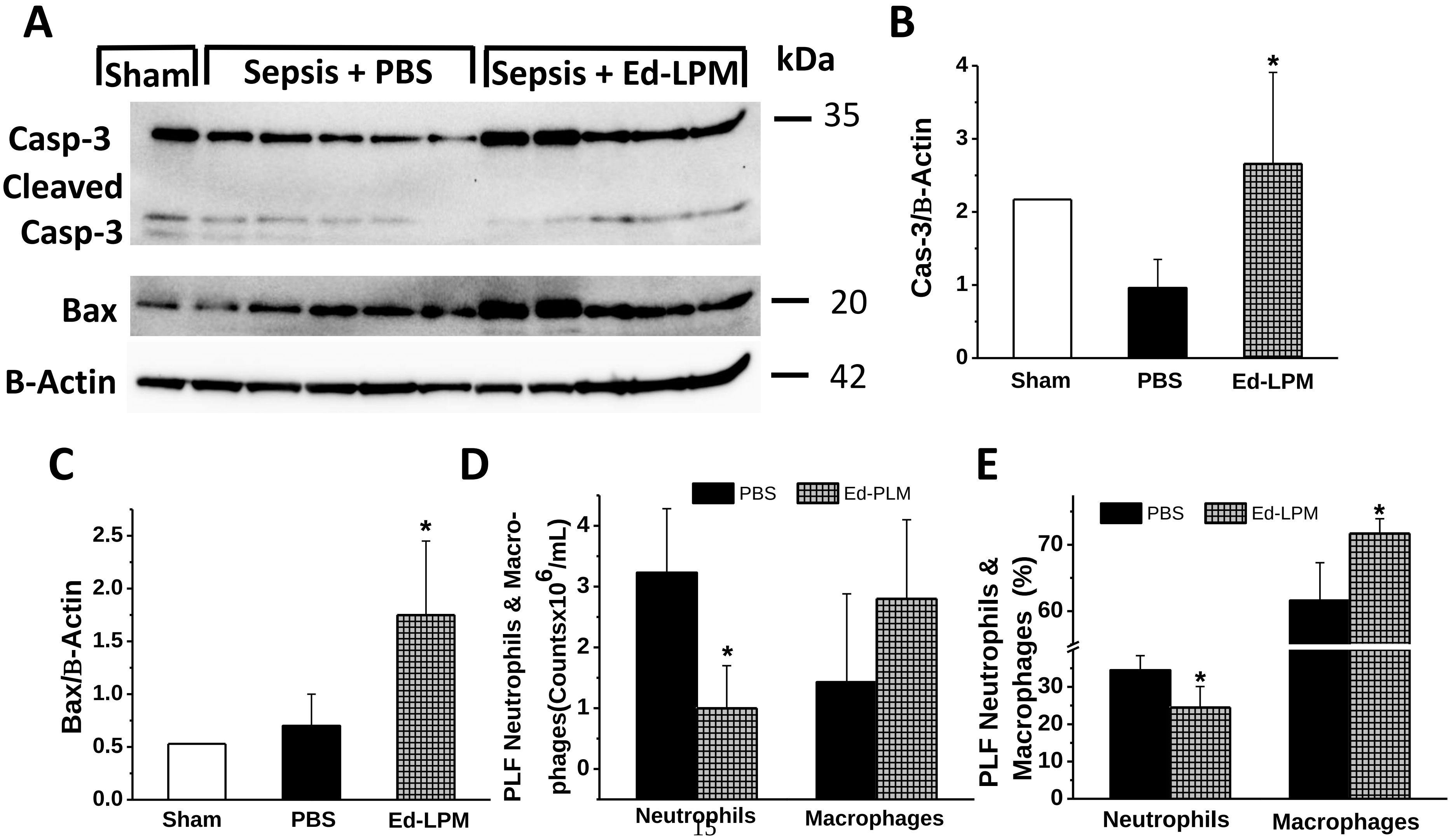

Figure S6. Ed-LPM therapy increases peritoneal neutrophil apoptosis and clearance

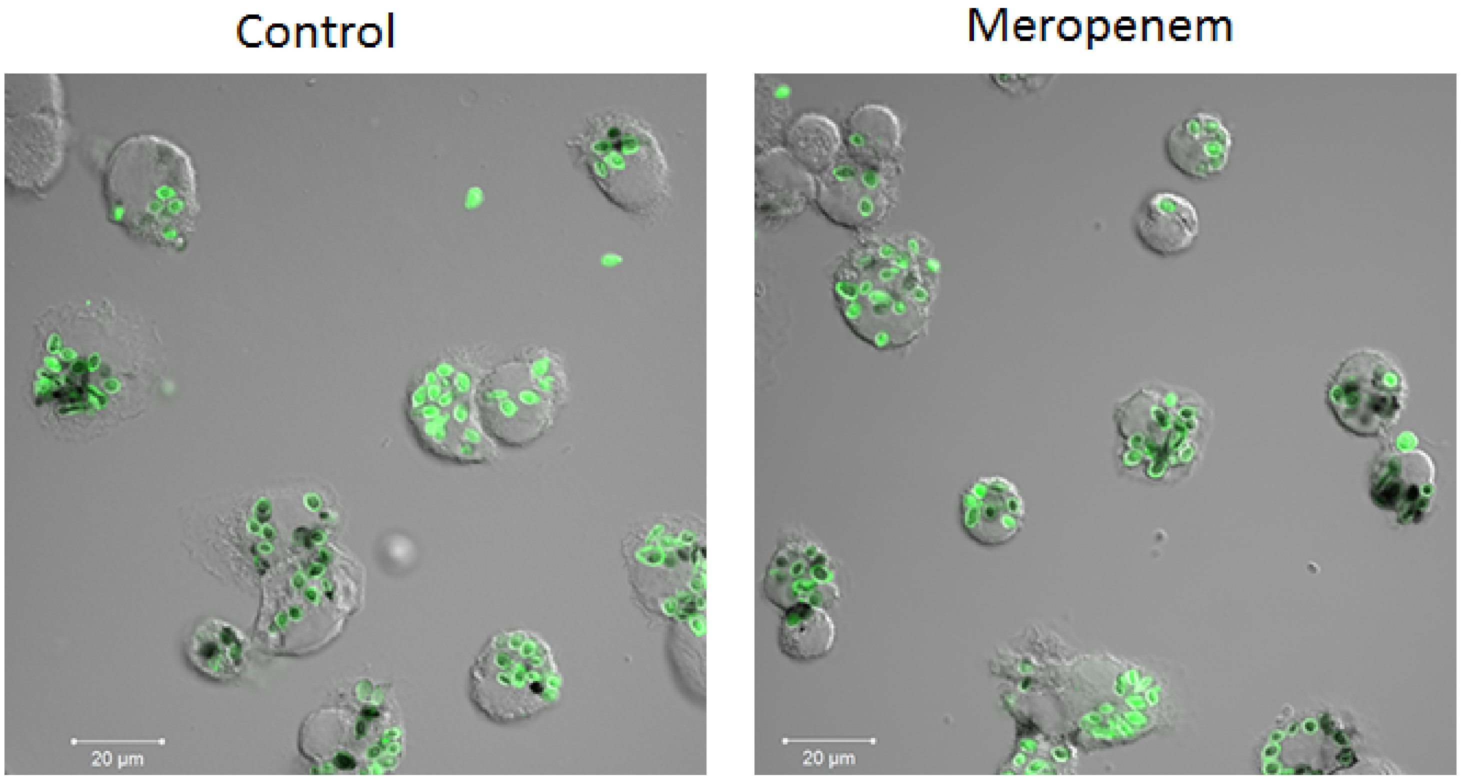

Figure S7. Meropenem did not modulate the function of rat Ed-LPMs

There was no difference in capabilities of peritoneal macrophages to phagocytose SOZ particles or to produce ROS following Meropenem exposure. To mimic *in vivo* experimental conditions and timing of Meropenem animal treatment, R-Ed-LPM were

seeded in 12-well plate (100,000/well) & treated with Meropenem (5mg/mL). Treatment was repeated 24h later. Next day (after 48h of Meropenem treatment) SOZ/NBT assay was done and confocal microscopy performed.

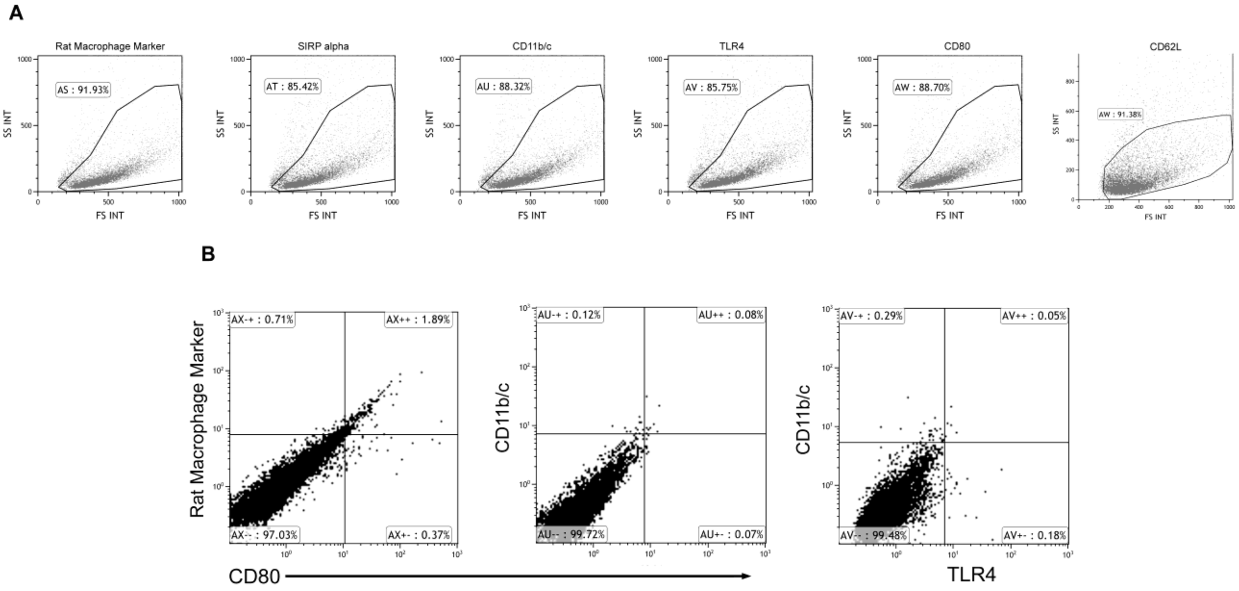

Figure S8: **Further characterization of Ed-LPMs.** (A) Representative base gates used for corresponding ligand markers indicated in figure 1B. (B) Representative negative gates used to define scatterplots presented in figure 1C. All acquisition was performed on a Becton Coulter bench top Gallios 10/3 using the manufacturer's recommendations as previously presented by Litvack and colleagues [1].

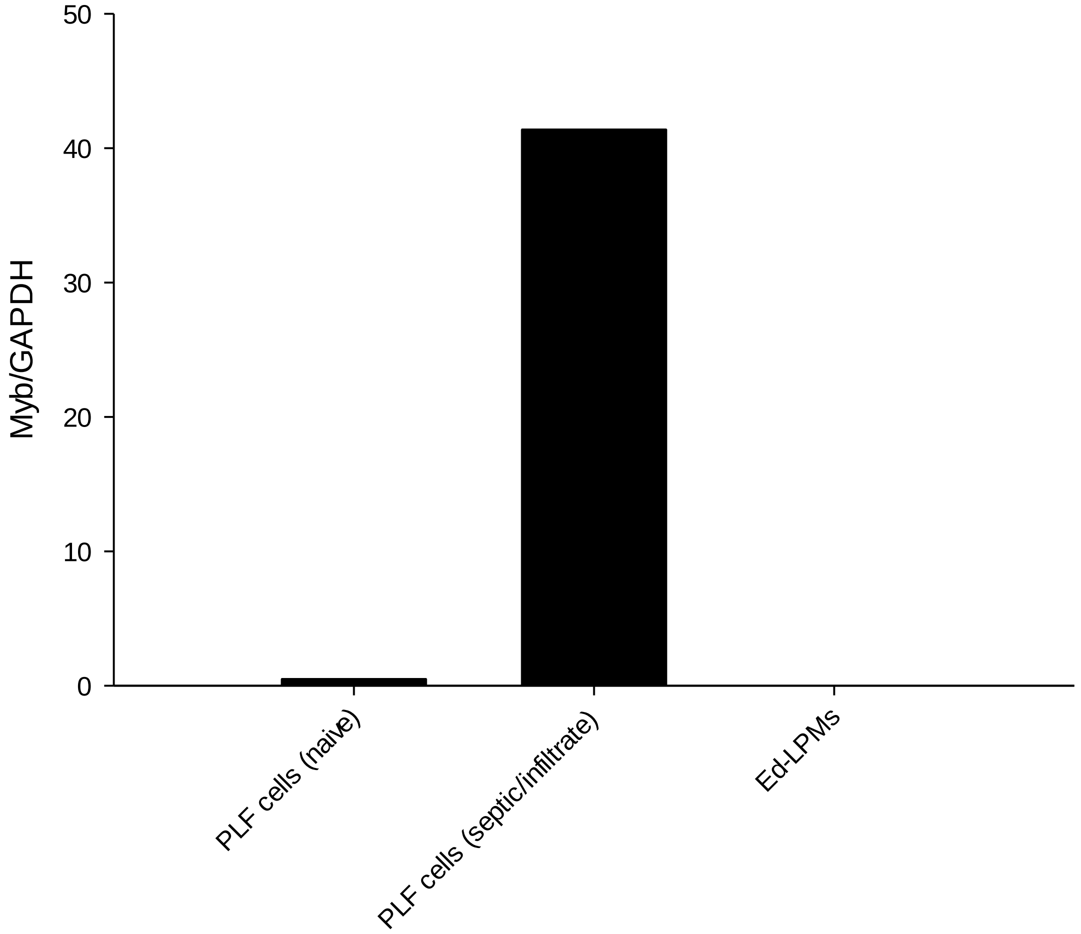

Figure S9. Expression of *Myb* in PLF cells and Ed-LPMs. Quantitative PCR was performed on PLF cells from naïve rats, septic rats and Ed-LPMs. The expression of *Myb* was compared as ratio of expression of the house keeping gene GAPDH.

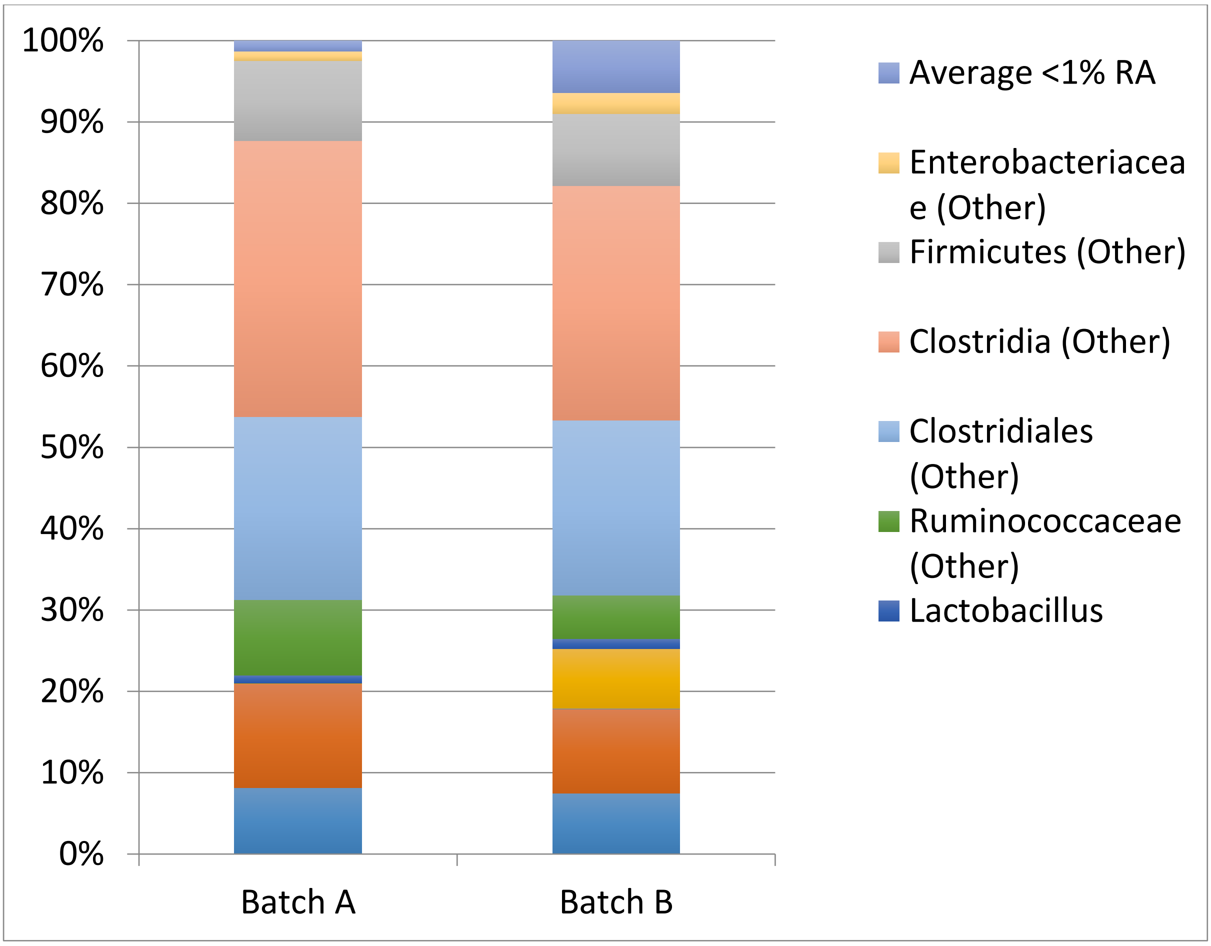

Figure S10. Differential microbial composition of two cecal slurry batches. Taxum genus showing difference between two slurry batches used in the experiments, which could account for different animal response and outcome of sepsis. Batch A was used in experimental series 1 and 2, while batch B was used in survival experiments with antibiotic Meropenem.

References

1. Litvack, M.L., et al., *Alveolar-like Stem Cell-derived Myb(-) Macrophages Promote Recovery and Survival in Airway Disease.* Am J Respir Crit Care Med, 2016. **193**(11): p. 1219-29.
2. Starr, M.E., et al., *A new cecal slurry preparation protocol with improved long-term reproducibility for animal models of sepsis.* PLoS One, 2014. **9**(12): p. e115705.
3. Costello, J., et al., *Hypercapnic acidosis attenuates shock and lung injury in early and prolonged systemic sepsis.* Crit Care Med, 2009. **37**(8): p. 2412-20.
4. Higgins, B.D., et al., *Differential effects of buffered hypercapnia versus hypercapnic acidosis on shock and lung injury induced by systemic sepsis.* Anesthesiology, 2009. **111**(6): p. 1317-26.
5. Nichol, A.D., et al., *Infection-induced lung injury is worsened after renal buffering of hypercapnic acidosis.* Crit Care Med, 2009. **37**(11): p. 2953-61.
6. O'Croinin, D.F., et al., *Sustained hypercapnic acidosis during pulmonary infection increases bacterial load and worsens lung injury.* Crit Care Med, 2008. **36**(7): p. 2128-35.
7. Rabani, R., et al., *Mesenchymal stem cells enhance NOX2-dependent reactive oxygen species production and bacterial killing in macrophages during sepsis.* Eur Respir J, 2018. **51**(4): p. pii: 1702021.
8. Canton, J., et al., *Contrasting phagosome pH regulation and maturation in human M1 and M2 macrophages.* Mol Biol Cell, 2014. **25**(21): p. 3330-41.
